# THE ASSOCIATION BETWEEN D3CR MUSCLE MASS AND MORTALITY IN COMMUNITY-DWELLING OLDER MEN

**DOI:** 10.1093/geroni/igz038.324

**Published:** 2019-11-08

**Authors:** Peggy M Cawthon, Terri Blackwell, Steven R Cummings, Eric S Orwoll, Kate A Duchowny, Kristine E Ensrud, Jane A Cauley, William J Evans

**Affiliations:** 1 California Pacific Medical Center Research Institute, San Francisco, California, United States; 2 Oregon Health & Science University, Portland, Oregon, United States; 3 University of California, San Francisco, San Francisco, California, United States; 4 University of Minnesota, Minneapolis, Minnesota, United States; 5 University of Pittsburgh, Pittsburgh, Pennsylvania, United States; 6 University of California, Berkeley, Berkeley, California, United States

## Abstract

We have shown that men with low muscle mass assessed by D3Cr (deuterated creatine) dilution are more likely to have worse physical performance and incident fractures, injurious falls and disability. However, the relation between D3Cr muscle mass and mortality is unknown. With data from Year 14 Visit of the MrOS study (N=1400, mean age 84.2 yrs), proportional hazards models estimated the risk of mortality (hazard ratio and 95% CI) by quartiles of D3Cr muscle mass (standardized to body mass); we calculated p for trend across quartiles. Models were adjusted for age, race, clinical center, alcohol use, smoking status, comorbidities, activity, percent fat, exhaustion, and cognitive function. Cause of death was centrally adjudicated. Over 3.3±0.8 years of follow-up, 197 (14.1%) men died. Men in the lowest quartile of D3Cr muscle mass/wgt were 2.8-fold more likely to die than men in the highest quartile (HR: 2.8, 95% CI: 1.6, 4.9; p for trend<.001). The HRs for each cause-specific mortality outcome were of similar magnitude to the HR for overall mortality: cancer death (HR, Q1 vs Q4: 2.2, 95% CI: 0.7, 7.1; p trend =0.140); CVD death (HR, Q1 vs Q4: 3.7, 95% CI: 1.3, 10.5; p trend =0.008); or non-cancer non-CVD death (HR, Q1 vs Q4: 2.4, 95% CI: 1.0, 5.6; p trend=0.019). We conclude that low muscle mass assessed by D3Cr dilution is a strong risk factor for mortality in older men, providing additional evidence that low muscle mass is an important risk factor for adverse health outcomes.

